# Strategies to improve dietary, fluid, dialysis or medication adherence in patients with end stage kidney disease on dialysis: A systematic review and meta-analysis of randomized intervention trials

**DOI:** 10.1371/journal.pone.0211479

**Published:** 2019-01-29

**Authors:** Karumathil M. Murali, Judy Mullan, Steven Roodenrys, Hicham C. Hassan, Kelly Lambert, Maureen Lonergan

**Affiliations:** 1 Department of Nephrology, Wollongong Hospital, Wollongong, NSW, Australia; 2 Centre for Health Research Illawarra Shoalhaven Population (CHRISP), University of Wollongong, Wollongong, NSW, Australia; 3 School of Psychology, University of Wollongong, Wollongong, NSW, Australia; University of Mississippi Medical Center, UNITED STATES

## Abstract

**Background:**

In patients with end stage kidney disease (ESKD) on dialysis, treatment non-adherence is common and results in poor health outcomes. However, the clinical benefits of interventions to improve adherence in dialysis patients are difficult to evaluate since trialled interventions and reported outcomes are highly diverse/ heterogeneous. This review summarizes existing literature on randomized controlled trials (RCTs) evaluating adherence interventions in ESKD patients focusing on the intervention category, outcome efficacy and persistence of benefit beyond the intervention.

**Methods:**

We performed electronic database searches in Medline, Embase & Cochrane CENTRAL upto 1^st^ July 2018 for RCTs evaluating interventions to improve diet, fluid, medication or dialysis adherence in ESKD patients. Study characteristics including category of interventions, outcomes, efficacy and follow-up were assessed. Meta-analysis was used to compute pooled estimates of the effects on the commonest reported outcome measures.

**Results:**

From 1311 citations, we included 36 RCTs (13 cluster-randomized trials), recruiting a total of 3510 dialysis patients (mean age 55.1 ± 5.8 years, males 58.1%). Overall risk of bias was ‘high’ for 24 and of ‘some concern’ for 12 studies. Most interventions (33 trials, 92%) addressed patient related factors, and included educational/cognitive (N = 11), behavioural / counselling (N = 4), psychological/affective (N = 4) interventions or a combination (N = 14) of the above. A majority of (28/36) RCTs showed improvement in some reported outcomes. Surrogate measures like changes in phosphate (N = 19) and inter-dialytic weight gain (N = 15) were the most common reported outcomes and both showed significant improvement in the meta-analysis. Sixteen trials reported follow-up (1–12 months) beyond intervention and the benefits waned or were absent in nine trials within 12 months post-intervention.

**Conclusions:**

Interventions to improve treatment adherence result in modest short-term benefits in surrogate outcome measures in dialysis patients, but significant improvements in trial design and outcome reporting are warranted to identify strategies that would achieve meaningful and sustainable clinical benefits.

**Limitations:**

Poor methodological quality of trials. Frequent use of surrogate outcomes measures. Low certainly of evidence.

## Introduction

Adherence to therapy, which is also known as treatment compliance, denotes the extent to which a person’s behaviour of taking medication, following a diet, and / or executing lifestyle changes, corresponds with the recommendations from a health care provider [1]. Poor adherence to treatment or treatment non-adherence is associated with worse health outcomes, in terms of increased mortality and morbidity [2]. However, non-adherence is common in patients with chronic diseases and patients with end stage kidney disease (ESKD) who are on dialysis are no exception [1]. Non-adherence may be intentional or un-intentional and several patient-related, disease-related, and treatment-related factors can contribute to non-adherence in dialysis patients [3].

Studies evaluating interventions to improve treatment adherence in dialysis patients have broadly addressed four domains of therapy; namely, adherence to recommendations regarding diet, fluid intake, dialysis treatment and medications [4, 5]. The lack of standardized methods to measure adherence in these domains, contributes to the reported variations in the rate of non-adherence, and the difficulty of precisely estimating the effectiveness of interventions to improve adherence [3, 5]. Methods of measuring adherence vary across studies and include indirect measures, such as self-reported adherence [6, 7]; direct measures such as pill counts or electronic medication event monitoring system (MEMS) [8], and attendance in dialysis sessions [9]; as well as surrogate measures such as inter-dialytic weight gain [10, 11] or biochemical parameters, which include phosphate and potassium levels [12, 13].

The interventions to help improve adherence in dialysis patients have also varied between studies. A systematic review of RCTs to improve adherence to dialysis, medication, diet and fluid intake in haemodialysis patients published in 2010, concluded that cognitive behavioural interventions offered the best promise for future studies [4]. A subsequent review by the same authors [5] and more recent systematic reviews focusing on specific outcome like inter-dialytic weight gain and phosphate control have included both randomized and non-randomized intervention studies [14, 15]. Non-randomized trials make up the majority of adherence intervention studies in dialysis patients [5], but the lack of random allocation of participants makes them susceptible to selection bias. Several pertinent randomized trials of adherence interventions have been published in the last decade, indicating a keen interest in this research area. In this context, we undertook a systematic review of RCTs in patients with ESKD undergoing dialysis (population), evaluating the effect of interventions to improve dietary, fluid, dialysis or medication adherence (intervention) compared to usual care or alternative strategies (control) on direct, indirect or surrogate measures (outcome) of adherence. Our objectives were to categorize various adherence interventions, examine whether the reported adherence outcomes are clinically meaningful, identify which interventions are effective in improving clinical outcomes and evaluate whether the benefits persist beyond the trialled interventions.

## Materials and methods

This systematic review, was structured on the Preferred Reporting Items for Systematic Reviews and Meta-Analyses (PRISMA) statement and check-list [16]. We included randomized trials published as full-text articles in the English language, which evaluated interventions to improve adherence to fluid, diet, medication or dialysis, or a combination of these domains, in ESKD patients undergoing dialysis. The review was registered at PROSPERO, the international prospective register of systematic reviews in February 2018 (http://www.crd.york.ac.uk/PROSPERO/display_record.php?ID=CRD42018087899)

### Search strategy

Electronic database searches were performed via OvidSP in the Medline, Embase and Cochrane central register of controlled trials for relevant articles using standard search strategies. Medical subject headings or search terms included combinations of ‘dialysis’, ‘renal dialysis’, ‘hemodialysis’, ‘peritoneal dialysis’, ‘patient compliance’, ‘adherence’, ‘medication adherence’, and text word searches using combinations of ‘adheren*’, ‘non-adheren*’ ‘nonadheren*’, ‘complian*’, ‘non-complian*’, ‘noncomplian*’, ‘fluid’, ‘diet’, ‘diet*’, ‘medication’, ‘dialys*’, inter-dialy*’, interdialy*’, ‘haemodialys*’, hemodialys*’, ‘peritoneal dialys*’, and ‘CAPD’ were conducted with searches restricted to ‘English’ and ‘humans’. An example search strategy used for Medline is provided as S1 Table. Search results in the form of titles and abstracts were analyzed by three authors (KM, HH, KL), to identify the studies to be included in the final review, based on the criteria outlined below. Any disagreement was resolved by discussion among all authors. In addition, references in the included articles and other important reviews on the topic were hand-searched to identify articles that might have been missed in the previous searches.

### Study selection criteria

Studies that evaluated adult ESKD patients undergoing haemodialysis or peritoneal dialysis were considered. Trials using random allocation of participants to different groups using a parallel group, cluster randomization or randomized crossover design were eligible for inclusion. Studies were included if they trialled at least one intervention, aimed at improving at least one measure of adherence pertaining to one or more domains of ESKD treatment adherence; namely, dietary, fluid, dialysis or medication adherence, as a pre-specified primary or secondary outcome. The reported measure of adherence outcome could have included indirect (e.g. self-reported adherence) or direct (e.g. MEMS-Medication event monitoring system), as well as surrogate measures, which included biochemical parameters (e.g. phosphate level) or inter-dialytic weight gain. For inclusion, studies needed to report the adherence measure before and after the intervention or the change in the adherence measure in response to the intervention. Non-randomized intervention trials and observational studies were excluded as were non-primary research articles (letters to the editor, brief communications and review articles).

### Data extraction and synthesis

A standard check-list developed by the authors was used to extract the following data from the included studies: the year of publication, journal, first author’s name, funding source, study design, number of participants in the intervention and control arms, study population characteristics, trialed intervention and control treatments, theoretical model of behaviour underpinning the intervention (if any), primary and secondary outcomes, measures of adherence before and after the intervention or the change in the adherence measures as a result of the intervention, whether adherence was directly measured during the conduct of the study, duration of follow-up, dropout rate, significant secondary outcomes and whether the benefits of intervention persisted on follow-up. If the intervention resulted in significant improvement in the pre-specified direct or indirect adherence efficacy measures (excluding knowledge), the study outcome was considered positive. However, if there was no significant improvement, the study was considered negative, or if there was improvement in some but not all of the pre-specified outcome measures, the study was considered partially positive. One author (KM) extracted the above information into the datasheet, and two authors (HH, KL) verified the accuracy.

A synthesis of the extracted data was then undertaken to group the various interventions under the broad categories of adherence interventions for chronic diseases proposed by the World Health Organization (WHO) [1], listed as (a) Social and economic interventions, (b) Health system / healthcare team related interventions, (c) Therapy related, (d) Condition or disease related and (e) Patient related interventions.

The patient level interventions were further sub-grouped into the categories of adherence interventions proposed by De Bleser et al [17]

1. Educational/cognitive interventions which provide information or knowledge about disease or treatment to the patient
2. Counselling/ behavioural interventions which addressed patient’s behaviour or skill relevant to self-care or empowered them to participate in their care
3. Psychologic/affective interventions that appealed to the patient’s feelings and emotions or social support and
4. Mixed interventions that involved a combination of the above-mentioned intervention types.

We also undertook meta-analysis to compute a pooled estimate of effect for the most commonly reported outcomes in the included trials.

### Quality of included studies

We assessed the risk of bias for the main outcome for each study, rather than applying a ‘quality scale’ to evaluate the methodological quality of the included trials. We opted against the ‘quality scales’ because they tend to combine and assign similar weighting to aspects of study conduct and quality of reporting, which is difficult to justify [18]. In this review we used the Cochrane risk of bias tool version 2.0 (ROB 2.0) for randomized trials [19] to assess study quality. Two authors (HH and KL) independently assessed the risk of bias for the five domains of potential bias using the ROB tool. The results were compared to reach a consensus on the risk of bias estimates by consultation and the remaining differences were resolved in discussion with the first author (KM). The overall risk of bias was assessed for each study in a similar fashion.

### Statistical methods

Continuous variables were expressed as means ± standard deviation and proportions were expressed as percentages. We compared proportions using Fisher’s exact test. Inter-rater reliability of the risk of bias domains was assessed using Cohen’s kappa statistics. In the meta-analysis, we used the mean difference in the adherence outcome between intervention and control arms as the effect measure, and the random effect option as the analysis model. When the standard deviation of the mean difference was not directly available for use in the meta-analysis from the publication, we contacted the authors seeking this information. However, if it was still unavailable, we computed standard deviation from the confidence intervals or p values cited in the paper, using t-statistics [20]. In situations where these metrics were not cited in the paper, we imputed the standard deviation from the arithmetic mean [20] of the standard deviations of the mean difference in the intervention and control arms respectively. Publication bias was evaluated using funnel plots along with the meta-analysis, which was conducted using Review manager software Version 5.3 (Copenhagen: The Nordic Cochrane Centre, The Cochrane Collaboration, 2014). Egger’s test was used to detect the skewness of the funnel plot and objectively assess the publication bias [21]. The statistical analyses were conducted using Stata version 15.1.

### Quality of evidence and ‘Summary of findings’ table

The quality of evidence in this systematic review was rated using the GRADE (Grade of Recommendation, Assessment, Development and Evaluation) approach, which takes into account various factors that can reduce the quality of evidence, such as within-trial risk of bias, inconsistency of results, indirectness of evidence, imprecision and publication bias, as well as factors that improve the quality like large magnitude of effect and dose-response gradient [22]. We used GRADEpro software to create a summary of findings table for the main outcomes, which provides an overall rating for each outcome and the explanations for grading the evidence [23].

## Results

### Search results

**Fig 1:**
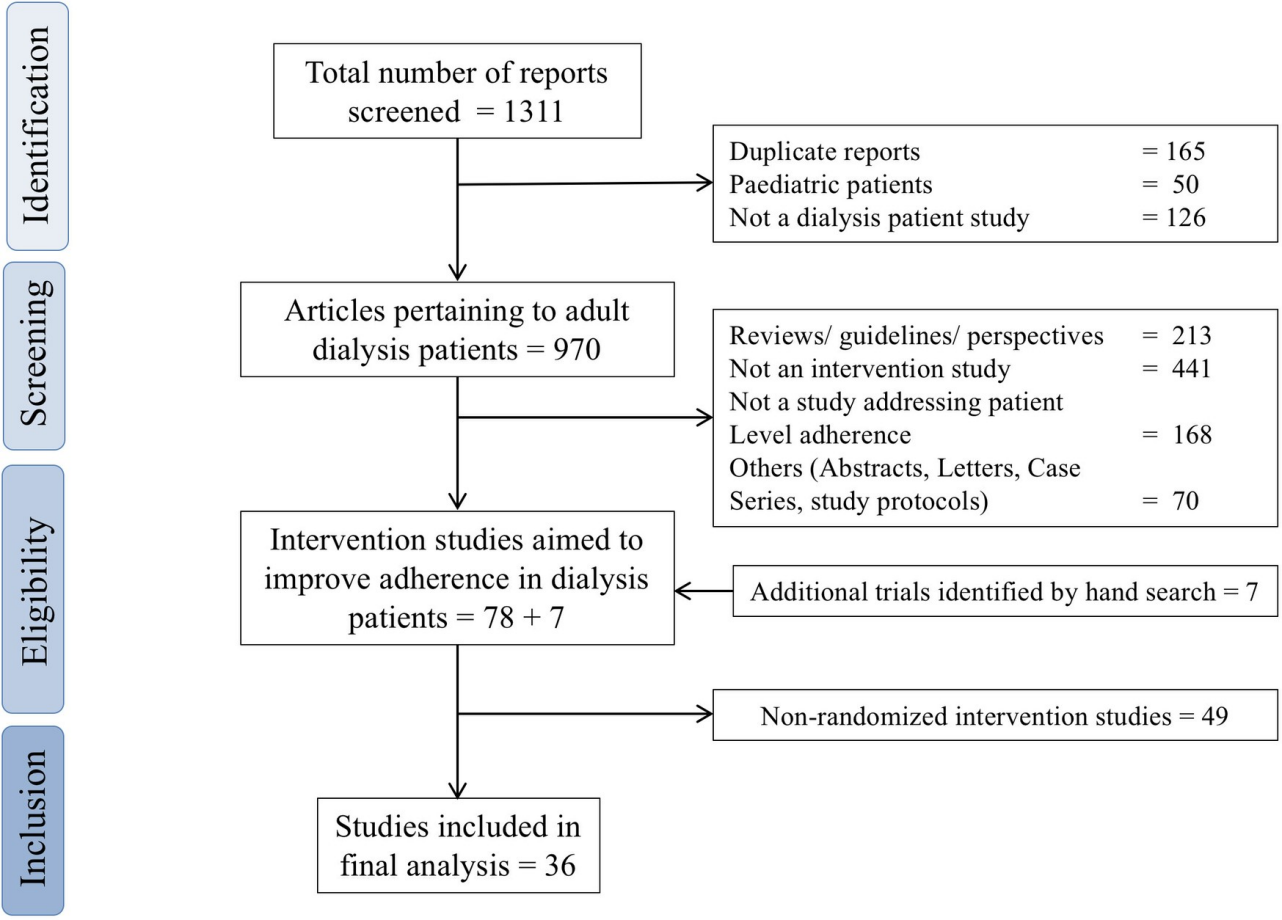
Flow chart of study selection in the systematic review.

Electronic searches in Medline, Embase and Cochrane central register of controlled trials were completed on 1^st^ July 2018 to identify relevant articles published till that date using search strategies described above. The searches did not limit the range of publication years. The broad electronic database search retrieved 1311 citations out of which 78 adherence intervention trials in dialysis patients were identified, based on the criteria outlined above. Seven trials, which were missed in the database search, were identified by hand search of references from important systematic reviews relevant to the topic. Out of these 85 studies, thirty-six randomized trials which fulfilled the specified inclusion and exclusion criteria were identified for inclusion in the review (for details, please refer to Fig 1).

### Study characteristics

Out of the 36 studies included in the review, 22 had a parallel group design, while one trial [10] adopted a randomized crossover design. The remaining studies were cluster randomized trials (for details please refer to Table 1).

**Table 1:** Characteristics of the trials included in the review.

Reference	Trial design / Population (Type of adherence) / Interventionist (theoretical models of behaviour if any)	Total No (Intervention/ Control) N/ Mean age (years)	Intervention	Domain and (category) of adherence intervention / Duration of intervention (d = day, wk = weeks, m = months)	Outcome relevant to treatment adherence	Total F/U in m(months) / Study result / Benefit sust-ained beyond intervention
Ashurst I de Brito et al (2003) [24]	Parallel group / HD (Diet + Medication) /Dietitian	58 (29/29) / 53.6	One-on-one education session by Dietitian based on "A Patient's guide to keeping healthy: Managing your phosphate", manual developed by Genzyme Pharmaceuticals. Also provided Medication chart to fill in self-administered doses of medications and blood results to increase patient engagement	Patient related interventions (Educational/cognitive) / 1 d	Change in levels of phosphate, calcium, calcium x phosphate product	6 m/ Partially positive
Baraz S et al (2010) [13]	Parallel group / HD (Diet+ Fluid+ Medication) / Nurses	63 (31/32) / 34.9	Oral education lasting 30 minutes each over two group sessions and a booklet "A patient's guide to controlling dietary regimen" vs Video education lasting 30 minutes during haemodialysis session with similar content including diet, importance of compliance etc	Patient related interventions (Educational/ cognitive) / 1 d	Change in IDWG. Change in levels of phosphate, calcium, potassium	4 m / Partially positive
Brantley P J et al (1990) [25]	Cluster randomized / HD (Vascular access cleansing) / Multiple staff	56 (14/14#/ 14#/14) / 56.6	Educational video of vascular access cleansing lasting 20minutes for 3 sessions over one week (Educational) / provision of visual cues to help cleaning, such as information board and monetary incentives in the form of raffles (Behavioural)/, the above two together (Educational & Behavioural) / attention control included video about vascular access without information on cleaning ((Control)	Patient related interventions (Educational/ cognitive & Counselling / behavioural) / 1 wk	Vascular access cleansing compliance	12 m / Positive at 1 month / Benefit wanes @ 12 m F/U
Chen W et al (2006) [26]	Parallel group / PD (Diet) / Dietitian	70 (35/35) / 55.3	Food menu suggestion and individualized education on food exchange based on patient preference	Patient related interventions (Educational/cognitive) / 1 d	Protein intake compliance computed from 3 days self-reported diet, Change in levels of phosphate, albumin	1 m / Partially positive
Cho M K et al (2013) [12]	Parallel group / HD (Diet + Fluid + Medication) / Nurses (King's theory of goal attainment)	43 (21/22) / 60.4	Health contract intervention lasting 30–60 minutes per week for 4 weeks which included formal introduction to the program, mutual goal setting, contracting and re-contracting to support selfcare behaviour reinforced through praise, encouragement and support	Patient related interventions (Counselling / behavioural) / 4 wk	Inventory (develop-ed by Song et al) to assess self-care behaviour including fluid intake, diet, medications, exe-rcise, physical man-agement and social adjustment, Change in IDWG, Change in levels of phosphate, potassium	1 m / Partially positive/ NA
Cukor D et al (2014) [10]	Crossover randomized / HD (Fluid) / Psychologist	59 (33/26)	Cognitive behavioural therapy (CBT) delivered chairside by psychologist over 60 minutes per for 3 months. Included psycho-education emphasising difference between depression and medical illness, components of adherence targeting dialysis compliance, adapting behavioural activation and identifying ESRD specific cognitive distortions	Patient related interventions (Educational/ cognitive & Counselling / behavioural) / 3 m	Change in IDWG	6 m / Partially positive / Positive @ 3 m/ Benefit wanes @ 3 m F/U
Cummings K M et al (1981) [27]	Parallel group / HD (Diet + Fluid) / Nurses (Health belief model)	96 (24/19#/ 28#/25) / 54.8	Three Intervention groups. 1. Behavioural contracting (included Identifying behaviour needing change, set time table for change, writing formal agreement and recording of progress) with reward schedule in the form of lottery tickets 2. Above intervention with family member/friend in addition to patient 3. Weekly telephone contact by nurse with structured content including identification of non-adherence, highlighting information on negative consequences of non-adherence and verbal support for maintaining adherence once a week for 6 weeks with hope of modifying health beliefs	Patient related interventions (Counselling / behavioural) / 6 wk	Change in IDWG. Change in levels of potassium, Health beliefs about diet and fluid	4.5 m / Partially positive / Positive @ 6 wk/ No benefit @ 3m F/U
de Araujo L P et al (2010) [28]	Parallel group / HD (Diet + Medication) / Multiple staff	33 (16/17) / 52.5	Six educational sessions lasting 30 minutes each about importance of avoiding high phosphate diet, correct use of phosphate binders, importance of blood results of calcium, phosphate, calcium, phosphate product, PTH and manifestations of bone disease in a course over 2 weeks	Patient related interventions (Educational/cognitive) / 2 wk	Change in levels of phosphate, calcium, calcium x phosphate product, PTH, Knowledge about diet and binders	3 m / Negative (only one outcome assessment @ 3 m)
Ford J C et al (2004) [29]	Parallel group / HD (Diet + Medication) / Dietitian	70 (35/35)	20–30 minutes of additional dietary education every month by dietician in addition to standard education, using tools including posters, handouts, puzzles, individual phosphorous tracking instruments highlighting dietary phosphate content, importance of diet, drugs and dialysis in phosphate control	Patient related interventions (Educational/cognitive) / 6 m	Change in levels of phosphate, calcium, calcium x phosphate product, PTH, Knowledge about diet and binders	6 m / Partially positive / NA
Forni Ogna V et al (2013) [8]	Parallel group / HD (Medication) / Multiple staff	50 (24/26) / 60.2	Integrated care approach (MEMS monitoring, motivational interviewing start 2m later with MEMS graphical report, identify barriers to non-adherence and strategies to address them, discussion about adherence)	Patient related interventions (Educational/cognitive & Counselling / behavioural) / 6 m	Change in levels of iPTH level, MEMS adherence (cinacalcet taking) and dose of Cinacalcet	9 m / Positive @ 6 m/ Benefit wanes @ 3 m post intervention
Griva K et al (2018) [30]	Cluster randomized / HD (Diet + Fluid + Med + Dialysis) / Multiple staff (Social cognitive theory)	235 (101/134) / 53.5	Three core and one booster group education sessions, totalling 8 hours, targeting self-management behaviour related to fluid intake, diet and medications and telephone follow-up between core & booster sessions	Patient related interventions (Educational/ cognitive & Counselling / behavioural) / 3 m (including core & booster sessions & phone F/U)	Change in IDWG. Change in levels of phosphate, potassium. Renal adherence behav-iour questionnaire (Fluid, potassium, phosphate, sodium, adherence in times of difficulty, self-care)	9 m / Positive @ 3m/ Benefit wanes @ 9 m post intervention
Haq N et al (2014) [31]	Parallel group / HD (Medication) / Dialysis staff	23 (12/11) / 52.5	Directly observed therapy in front of haemodialysis nurses administering cinacalcet 3 times a week during dialysis	Health system related interventions (Supervised therapy) / 4 m	Change in levels of phosphate, calcium, PTH	4 m / Negative / NA
Hare J et al (2014) [32]	Parallel group / PD (Fluid) / Psychologist (Health belief model)	15 (8/7) / 60.1	Group format CBT for groups of 6–8 patients at a time in 1-hour sessions per week for 4 weeks. The program was called Liquid intake program (LIP) which was adapted (and renamed) from the Glasgow university liquid program (GULP) used in by Sharp et al 2005. The structured program included Introduction, goal setting and environment change, thought, emotions and behaviour as well as social support and program review	Patient related interventions (Educational/ cognitive & Counselling /behavioural) / 4 wk	Fluid adherence assessed by weight reduction >2Kg, BP, Psychological markers (psychological well-being, quality of life, health beliefs)	2.5 m / Negative post-intervention & 6 wk F/U
Hou Y M et al (2010) [33]	Parallel group / HD (Fluid) / Psychologist (ABC theory)	92 (48/44) / 44.6	Rational emotive therapy establishing a good patient-caregiver relationship with a structured program with introduction providing basic knowledge and psychological health, description of rational emotive therapy and ABC theory, procedure of rational emotive therapy in three phases including psycho-diagnosis, comprehension and application	Patient related interventions (Psychological / affective) / 3 m	Change in IDWG, BP, Ultrafiltration volume	3 m /Positive / NA
Howren M B et al (2016) [34]	Cluster randomized / HD (Fluid) / Psychologist (Self-regulation theory)	119 (61/58) /57.1	Highly structured behavioural self-regulation intervention sessi-ons administered by psychologists to group of 3–8 participants lasting one hour weekly for 7 weeks, including illustration of behavioural principles, group discussions and homework assign-ments specific to fluid adherence comprising of self-regulation techniques, goal setting, self-administered reinforcement strategies, stimulus control, evaluation of group experience	Patient related interventions (Counselling /behavioural & Psychological / affective) / 7 wk	Change in IDWG	8 m / Positive / Benefit present @ 6 m F/U
Karavetian M et al (2013) [7]	Cluster randomized / HD (Diet + Medication) / Dietitian (Self efficacy theory)	122 (41/41 #/40) / 57.0	Self-management dietary counselling and interactive games for 20 minutes per week for 8 weeks. Also included discussion for 10 minutes every month about bone mineral disease related parameters and relevant nutritional counselling	Patient related interventions (Educational/ cognitive & Counselling / behavioural) / 8wk	Change in levels of phosphate, calcium, calcium x phosphate product. Knowledge. Dietary non-adherence by questionnaire	2 m / Positive / NA
Karavetian M et al (2015) [35]	Cluster randomized / HD (Diet) / Dietitian (Trans-theoretical model)	394 (88/ 201# /96)/ 58.8	Individualized intensive trans-theoretical stage-based nutrition education twice weekly for six months by an academic dietitian. A partial intervention group provided a second control group	Patient related interventions (Educational/cognitive) / 6 m	Change in phosphate levels. Phosphate intake. Knowledge about dietary phosphate	12 m / Positive at 6 month / Benefit wanes @ 6 m F/U
Kauric-Klein Z et al (2012) [9]	Cluster randomized / HD (Diet + Fluid + Medication + Dialysis) / Nurses	118 (59/59) / 59.7	Two blood pressure education sessions, self-monitoring of BP for 12 weeks checking it twice a day with logs, goal setting for BP levels, Fluid gains and Salt intake and haemodialysis compliance with reinforcement sessions lasting 10–15 minutes per week for twelve weeks with supportive nursing intervention	Patient related interventions (Educational/ cognitive & Counselling / behavioural) / 12wk	BP self-care beh-aviour, (IDWG, Salt intake, BP med adherence–self-re- ported), BP, Dialysis adherence (missed HD sessions)	4 m /Partially positive @ 3m / Benefit persists @ 1 m F/U
Lou LM et al (2012) [36]	Cluster randomized / HD (Diet) / Dietitian	80 (41/39) / 62.3	Register with dietitian who provides detailed menu suggestions adapted to patients plus targeted dietary education concerning phosphorous intake lasting 30minutes every by dietitian	Patient related interventions (Educational/cognitive) / 6 m	Change in levels of phosphate. Per-centage of patients achieving phos-phate goal. PTH. Nutritional measur-es—BMI/ albumin/ fat free mass	6 m / Positive / NA
Molaison E F et al (2003) [37]	Cluster randomized / HD (Fluid) / Multiple staff (Trans-theoretical model)	316 (216/100) / 53.8	Dietitian intervention using stages of change in the trans-theoretical model to improve fluid intake. First 6 weeks phase of Pre-action including precontemplation and contemplation, second 6week phase of Action including preparation, action and maintenance. Communication to patients maintained through bulletin boards, handouts and feedback. Constructs included consciousness raising, self-evaluation, counter-conditioning, stimulus control, self-efficacy etc	Patient related interventions (Educational/ cognitive & Counselling / behavioural) / 12wk	Change in IDWG, Stage of change in fluid adherence, Knowledge	3 m / Negative /Knowledge improved / NA
Morey B et al (2008) [38]	Parallel group / HD (Diet + Medication) / Dietitian	67 (34/33) / 57.7	Intensive dietary counselling every month for 6months about phosphate in diet and phosphate binder use using motivational counselling, behaviour modification therapy, reminders, reinforcement, supportive care as well as written and verbal education	Patient related interventions (Educational/ cognitive & Counselling /behavioural) / 6 m	Change in levels of phosphate, calcium, calcium x phosph-ate product, PTH, albumin, Nutritional measures: hand grip, mid-arm circumference	12 m / Negative after intervention @ 6 m & 6 m F/U
Neumann C L et al (2013) [11]	Parallel group / HD (Fluid) / Multiple staff	120 (60/60) / 66.1	Body weight telemetry with phone calls triggered by thresholds detected during monitoring with >1.5kg/d weight gain mandating phone call, 0.75–1.5Kg/d weight gain prompting individualized decision making based on patient profile	Health system related interventions (Monitoring / engagement) / 3 m	Change in IDWG, BP	3 m / Partially positive / NA
Pasyar N et al (2015) [39]	Parallel group / HD (Diet + Fluid + Medication) / Professional relaxation therapist	86 (43/43)	Benson relaxation technique of progressive muscle relaxation with breathing awareness for 20 minutes twice a day for 8 weeks	Patient related interventions (Psychological / affective) / 8 wk	Change in IDWG, Change in levels of phosphate, potassium, chemistry	2 m / Partially positive / NA
Reese P P et al (2015) [6]	Parallel group / HD (Diet + Medication) / Dietitian	36 (12/12#/ 12) / 53.0	Intervention group 1 received financial incentives including envelopes with cash and lottery for achieving goal range and those with above goal range received envelopes with messages designed to avoid regret aversion. Intervention group 2 received coaching for 45–60 minutes 3 times a week by Dietitian trained in motivational interviewing, structured as per Precaution Adoption process model in addition to discussing phosphate in diet, phosphate binders and identified personalized goals to achieve dietary and medication adherence	Patient related interventions (Educational/ cognitive & Counselling / behavioural) / 10 wk	Change in level of phosphate, Medication adherence by questionnaire	2.5 m / Negative / NA
Sehgal A R et al (2002) [40]	Cluster randomized / HD (Dialysis) / Multiple staff	169 (85/84) / 54.5	Identify barriers with respect to low prescription, catheter use for access and shortened treatment time & individually address by liaising with randomized nephrologists and engaging with patients to resolve barriers	Health system related interventions / Patient related interventions (Monitoring / engage-ment & Educational / cognitive) / 6m	Change in Kt/V, Proportion of patients achieving goal Kt/V	6 m / Positive / NA
Sharp J et al (2005) [41]	Cluster randomized / HD (Fluid) / Psychologist (Health belief model)	56 (29/27) / 54.3	Glasgow University liquid intake program (GULP) administered by Psychologist structured as small group (3–8 subjects) interactive sessions lasting 1 hour per week for 4 weeks, with educational component providing information about importance of fluid restriction, behavioural component of teaching self-monitoring skills, goal setting and self-regulation as well as cognitive components of encouraging to identify association between thoughts, emotions and behaviours. Patients were advised to complete thought records	Patient related interventions (Educational/ cognitive & Counselling / behavioural) / 4 wk	Change in IDWG, Health-belief Questionnaire adapted from Friend et al regarding fluid	3.5 m / Negative @ 4 weeks / Benefit within group @ 10 wk F/U
Shi Y X et al (2013) [42]	Parallel group / HD (Diet + Medication) / Nurses	80 (40/40) / 53.3	Nurse led education lasting 30 minutes two or three times a week for six months, written educational material and monthly group educational sessions for six months	Patient related interventions (Educational/cognitive) / 6 m	Change in levels of phosphate, calcium, calcium x phosphat- e product, albumin, Knowledge	6 m / Positive / NA
Skoutakis V A et al (1978) [43]	Parallel group / HD (Diet + Medication) / Pharmacist	24 (12/12) / 47.0	Pharmacist review two to three times a week for four months supplying educational materials, consultation regarding health, benefits of compliance with diet and medicines and written reminders about taking oral medications. Clarification of physician instructions and drug titration advice was also provided	Patient related interventions (Educational/ cognitive) / 4 m	Knowledge, Weighted drug dose compliance (differ-ent drug classes). Weighted biochem-ical profile (potassium, urea, Weight gain, BP)	8 m / Positive / Benefit wanes @ 4 m F/U
Sullivan C et al (2009) [44]	Parallel group / Cluster randomized / HD (Diet) / Dietitian	279 (145/134) / 53.0	Education about phosphorous content of food additives, provision of magnifier lens to enable food label readings, printed information containing fast food info and better choices. Telephone contact next month to reinforce advice	Patient related interventions (Educational/ cognitive) / 2 m (including telephone F/U)	Change in level of phosphate, Nutritional knowledge, Reading labels	3 m / Partially positive / NA
Tanner JL et al (1998) [45]	Parallel group / HD (Diet + Fluid + Medication) / Dietitian (Health belief model / Self efficacy theory)	40 (30/10) / 50.2	Monthly progress report with IDWG (<3Kg weekdays, <4Kg weekends), and phosphorous (<5.9mg/dl) goals with sticker incentives for acceptable results and monthly written behavioural contracts to assist in one or more goals. Results reviewed monthly and recontracted each month, increasing complexity over time	Patient related interventions (Counselling / behavioural) / 6 m	Change in level of phosphate, Number of dialysis sessions with acceptable IDWG (>8 of 12 HD sessions), Knowle-dge, Self-efficacy	6m / Negative (Knowledge was better @ 6 m) / NA
Tsay S L et al (2003) [46]	Parallel group / HD (Fluid) / Nurses (Self efficacy theory)	62 (31/31) / 57.7	Structured self-efficacy training comprising of a total of 12 sessions of one hour each, individualized education about renal failure, haemodialysis, medications, fluid restrictions performance mastery, realistic gaol setting, verbal persuasion with encouragement and decreased arousal through physical relaxation listening to audiotapes. Patients also were advised to maintain food and fluid records	Patient related interventions (Educational /cognitive & Counselling /behavioural) / 1 m	Change in IDWG	6m / Positive / Benefits present @ 6m
Welch J L et al (2013) [47]	Parallel group / HD (Fluid + Overall) / Nurses (Social cognitive theory)	44 (24/20) / 50.3	Dietary intake monitoring application (DIMA) a mobile application developed using nutrition database and universal product code (UPC) database, provides individualized ongoing information to assist patients with dietary and fluid self-monitoring	Patient related interventions (Educational/cognitive & Counselling /behavioural) / 6 wk	Change in IDWG, Self-efficacy measures by modified Cardiac diet self-efficacy (SE) instrument & Fluid SE scale	14 wk / Negative / NA
Wileman V et al (2016) [48]	Cluster randomized / HD (Fluid) / Psychologist (self-affirmation theory)	89 (49/40) / 60.7	Re-affirmation act before receiving health information about fluid overload and risks at baseline and briefer re-affirmation act before health information at 1,3 and 6 months. The act required participants to recall past act of kindness. The health information was followed by questionnaire exploring perception of risk, intention and self-efficacy to control their fluid intake.	Patient related interventions (Psychological / affective) / 6m (including F/U sessions @ 3m & 6m)	Change in IDWG. Self-reported measure of fluid intake (1–5 scale), Self-efficacy	12 m / Partially positive / Benefits persisted @ 12 m
Wileman V et al (2014) [49]	Cluster randomized / HD (Diet + Medication) / Psychologist (self-affirmation theory)	112 (57/55) / 60.5	Re-affirmation act before receiving health information about phosphate control and risks at baseline and briefer re-affirm-ation act before health information at 1,3 and 6 months. The act required participants to recall past act of kindness. The health information was followed by questionnaire exploring perception of risk, intention and self-efficacy to control their phosphate.	Patient related interventions (Psychological / affective) / 1 d	Change in phosphate levels, Self-reported measures, Self-efficacy	12 m / Partially positive / Benefits persisted @ 12 m
Wong F K Y et al (2010) [50]	Parallel group / PD (Diet + Fluid+ Medication + Dialysis) / Nurses	98 (49/49) / 62.4	Nurse led disease management program for 6 wk based on the 4-Cs model comprising of Comprehensiveness, Collaboration, Coordination and Continuity-run by Renal & General nurses. Content of the program included assessment using the Omaha system modified for renal patients, arts and skills of telephone nursing, setting mutual goals and health coaching, use of disease management protocols and concept of disease management process and outcomes	Health system related interventions (Monitoring / engagement) / 6 wk	Self-reported adherence using a modified version of dialysis, diet and fluid questionnaire (DDFQ)	3 m / Partially positive / Some benefits persist @ 3m
Yokum D et al (20018) [51]	Parallel group / HD (Diet + Medications) / Multiple staff	34 (17/17) / 49.4	Pharmacist & Dietitian adjust phosphate binders as per protocol in addition to monthly reviews by Pharmacist and Dietitian to provide education and reinforcement	Health system related interventions / Patient related interventions (Monitoring / engagement & Educational / cognitive)	Change in phosphate, calcium, calcium x phosphate product, PTH	4 m / Partially positive / NA

Abbreviations or symbols used in the table: BP = blood pressure, CBT = cognitive behavioural therapy, ‘d’ = day, F/U = follow-up, HD = haemodialysis, IDWG = inter-dialytic weight gain, ‘m’ = months, MEMS = medication event monitoring system, NA = not applicable, PD = peritoneal dialysis, PTH parathyroid hormone, ‘wk’ = weeks,

# indicates the number of patients in alternative intervention group. Additional details about the study characteristics and outcome data are provided in S2 Table.

The total number of participants in the included studies was 3510, with 1729 in the intervention and 1781 in the control arms. The number of participants in the different trials ranged from 15 to 394 [32, 35] with a median of 70 patients. Three trials recruited patients undergoing peritoneal dialysis [26, 32, 50] while the remaining 33 studies recruited haemodialysis patients. The mean age was 55.1years (standard deviation (SD) 5.8years) and male patients constituted a majority (mean 58.1%, SD 12.2%) of the study participants.

Four studies [8, 11, 24, 51] indicated that they were partly or fully supported by pharmaceutical sponsors, whereas 20 studies were funded by public organisations, including universities. No information on funding source was provided in twelve studies.

### Risk of bias

**Fig 2:**
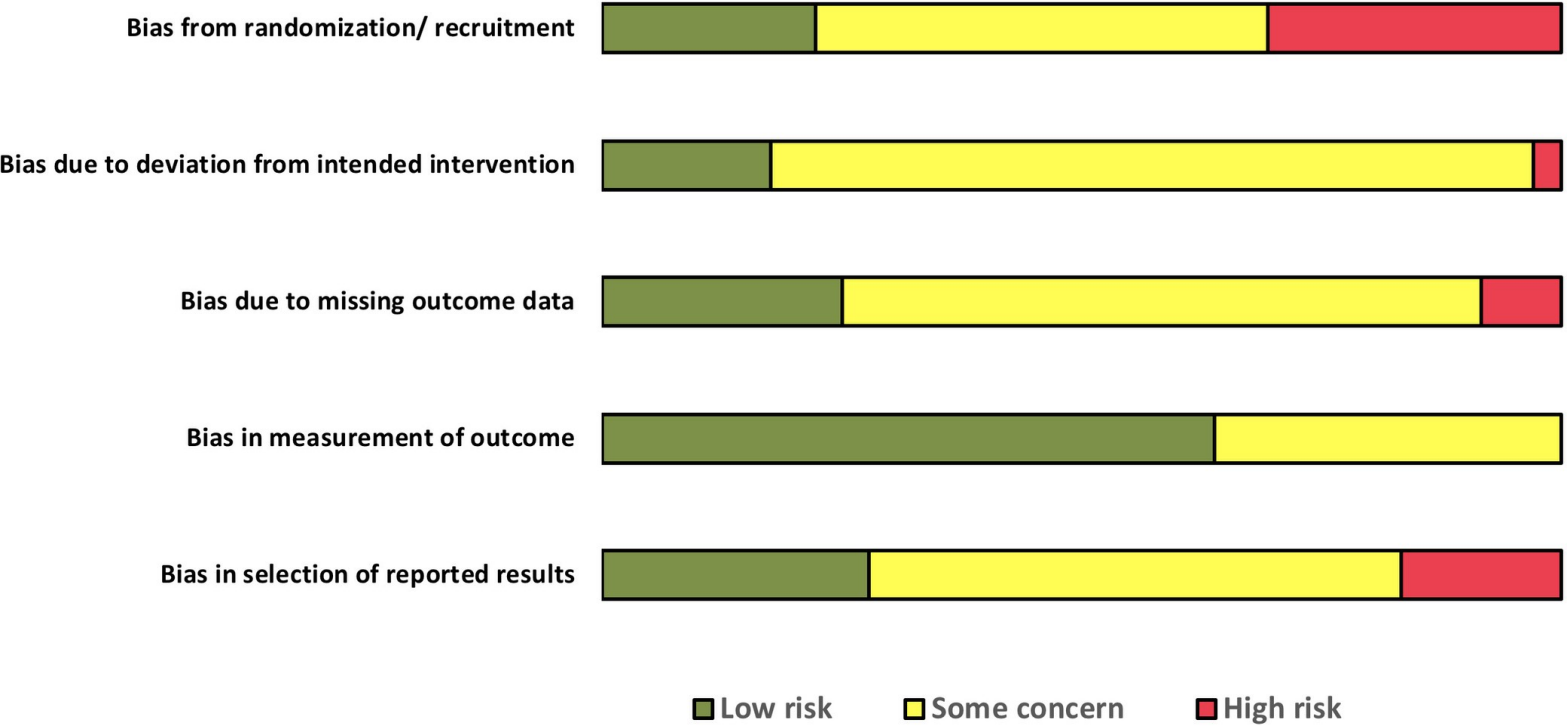
Risk of bias assessment of the included studies. The data presented for individual risk of bias domains are based on Cochrane ROB 2.0 tool [19] for randomized trials.

Assessment of included studies using the Cochrane ROB 2.0 tool [19] with respect to the five domains of potential risk of bias, showed a high inter-rater agreement of 76.7% (between authors HH and KL) based on independently abstracted data (Kappa 0.58, p <0.001), which was further strengthened (inter-rater agreement 95.6%, Kappa 0.92, p <0.001) after consultation. The remaining differences were resolved by discussion between authors. The overall risk of bias in the included trials was judged as ‘high risk’ for 24 studies and ‘some concern’ for the remaining 12 studies. With respect to four out of the five individual risk domains, a majority of the studies were judged to be of ‘some concern’ or ‘high risk’ (for details refer to Fig 2). With respect to the ‘risk of bias in measurement of the outcome’, even though blinding of the outcome assessment was not implemented or specified in most of the studies, more than half of the included trials were assessed as ‘low risk’. This was because, the main efficacy measures in these trials were biochemical measurements or changes in body weight, which may not be impacted by the lack of blinding of the outcome assessor. It should be appreciated that these surrogate adherence outcomes are susceptible to measurement error, depending on the test conditions. Changes in the timing of blood sampling in relation to dialysis, length of inter-dialytic interval, or variations in clothing worn by the subject can lead to biased measurement of phosphate levels or body weight. However, such variations will not be modified by outcome assessor blinding and have not been factored into the risk of bias estimates in this review. Additional details of the risk of bias assessment of individual studies are provided in S3 Table.

### Interventions

When the article did not describe which aspect of ESKD treatment adherence, -i.e. dietary, fluid-related, dialysis related or medication adherence, was addressed in the study, it was inferred from the nature of the trialled interventions or the reported outcome. For example, reporting of inter-dialytic weight gain as an outcome was interpreted as evaluating adherence to fluid recommendations, while changes in phosphate level as an outcome were interpreted as testing adherence to dietary and medication recommendations. It is acknowledged that these assumptions may not be valid under all circumstances and could lead to misclassification in some cases. Fluid adherence was assessed in nine studies, medication adherence tested in two studies [8, 31], while four studies [26, 35, 36, 44] assessed dietary adherence, and one study each evaluated dialysis adherence [40] and vascular access cleansing [25]. The remaining nineteen studies evaluated various combinations of dietary, medication, dialysis and fluid adherence (refer to Table 1).

The evaluated intervention was delivered by a variety of health care professionals in the different studies, with dietitians (10 trials) and nurses (9 trials) being the most frequent. Psychologists were the interventionists in seven trials, while a pharmacist [43] and relaxation therapy professional [39] delivered the intervention in one study each. The remaining eight studies had multiple staff involved or provided no specific information on the interventionist.

In fifteen studies (42%), theoretical models of behaviour relevant to treatment adherence formed the basis of the trialled intervention. The health belief model [27, 32, 41, 45] was the most commonly used, while self-efficacy theory [7, 45, 46], social cognitive theory [30, 47], self-affirmation theory [48, 49], trans-theoretical models (TTM) [35, 37], self-regulation theory [34], King’s theory of goal attainment [12] and the ABC (Antecedents, Behaviour, Consequences) model relevant to rational emotive therapy [33] were also invoked. Six out of seven studies, where the interventionist was a psychologist, had a theoretical behavioural underpinning.

Taking into account the five categories of adherence interventions outlined in the WHO report [1], 33 studies (92%) addressed patient related factors. Health system or health care team related interventions were tested in five studies [11, 31, 40, 50, 51], two of which [40, 51] also addressed patient related factors. Interventions addressing social-economic factors, therapy related factors or disease or condition related factors were not tested in any of the trials (Refer to Table 1).

When we assigned the patient related interventions into the subcategories proposed by De Bleser et al [17], several studies appeared to align with more than one subcategory. The dominant category was assigned by consensus, with guidance when necessary from the senior psychologist among the authors. Eleven studies evaluated educational or cognitive interventions, four had behavioural or counselling interventions [6, 12, 27, 45], four had psychological or affective interventions [33, 39, 48, 49] and fourteen studies had elements of different categories in the trialled intervention (refer to Table 1).

### Controls

Twenty six of the 36 randomized trials (72%) assigned control patients to usual or standard care, including standard health or nutritional education. Wait-listed controls were employed in three studies [10, 32, 41], while the remaining studies used some type of intervention as a comparator to match the active intervention. The comparator included attention control [25], placebo support and discussion control conditions [34], provision of matching health information without prior reaffirmation activity [48, 49], use of a daily activity monitoring application [47], and health education which was different from the trialled intervention [13, 28]. Four studies [7, 25, 27, 35] included in this review also had two or more active (partial or alternative) intervention arms in addition to the main intervention and control arms (refer to Table 1).

### Outcome assessment

The reported outcome data which reflected treatment adherence were diverse and mostly included surrogate measures (refer to Table 1). For the only crossover study [10] in our review, we included the data for the initial phase of randomized comparison between the intervention and control arms, before the crossover. Inter-dialytic weight gain or change in weight or proportion of sessions with satisfactory weight gain was reported as a fluid adherence outcome in 18 studies, while blood pressure (BP) control was assessed in four studies. Biochemical parameters were reported as an adherence outcome in 23 studies, which included combinations of blood levels or change in levels of phosphate (n = 19), calcium (n = 10), calcium phosphate product (n = 7), PTH (n = 7), potassium (n = 5) or albumin (n = 3). Dialysis adherence data, identified as missed or shortened dialysis sessions or changes in biochemical parameters (pre-dialysis BUN, Kt/V), was provided in three studies[30, 40, 43]. Dietary adherence information was provided in two studies [9, 26]. Indirect estimates of adherence, as self-reported or otherwise, were given in seven studies, while direct measures of medication adherence using an electronic medication event monitoring system (MEMS) was reported in only one study [8]. Assessment of knowledge or beliefs about disease or therapy, including medications, or self-efficacy was reported in six studies (refer to Table 1).

### Outcome efficacy

The trialled intervention was effective in improving the adherence outcome measures in twelve studies, while in sixteen studies the intervention led to mixed results, with improvement in some pre-specified outcome measures but failing to show significant benefit in other adherence outcomes (refer to Table 1). For example, in twelve trials which reported two or more biochemical markers as surrogate outcome measures in response to adherence interventions, eight showed improvement in phosphate levels, but five of these studies [13, 24, 29, 39, 51] showed no significant improvement in other surrogate adherence outcomes such as calcium x phosphate product, PTH or potassium. The evaluated intervention, did not result in an improvement in any of the adherence outcome measures in eight trials, although two of these studies [37, 45] showed improvement in the participant’s knowledge and awareness about the relevant issues, as a result of the intervention (refer to Table 1). The outcome efficacy, whether positive or negative, was not significantly associated with the type of interventionist (dietitian or nurses or others) (p = 0.929, Fisher’s exact test), the category of intervention (educational/cognitive or others) (p = 0.388, Fisher’s exact test) or the use of a theoretical model of behaviour (p = 0.694, Fisher’s exact test) in planning the intervention.

### Meta-analysis

**Fig 3:**
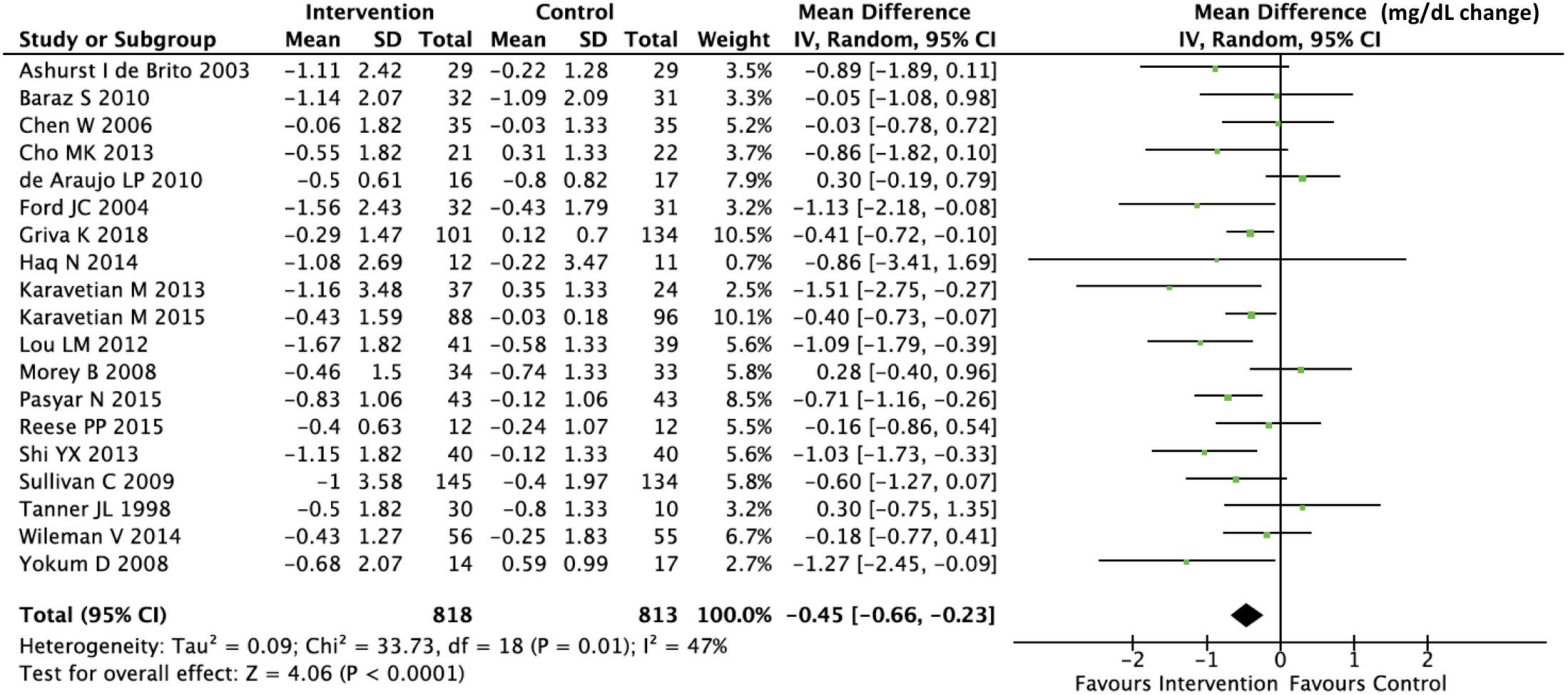
Forest plot showing the change in phosphate in response to adherence interventions. The mean difference is measured in mg/dL (to convert mg/dL to mmol/L, multiply by 0.323).

**Fig 4:**
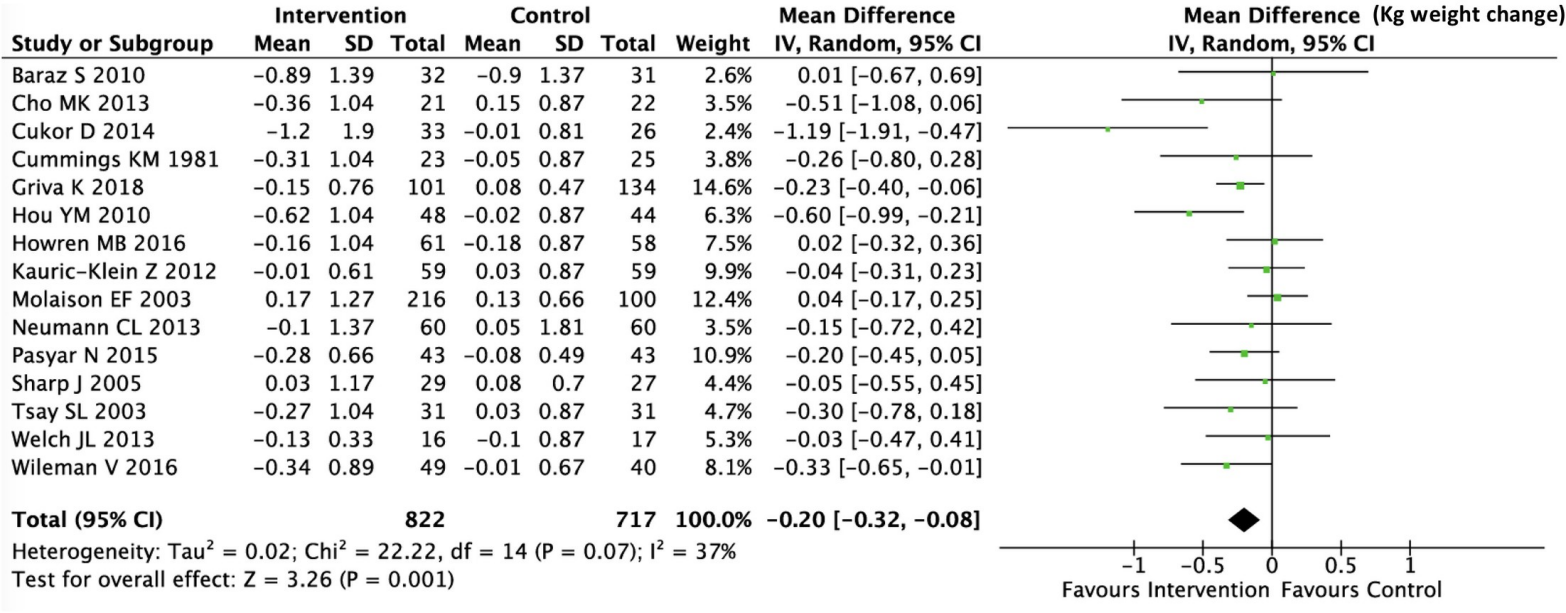
Forest plot showing the change in inter-dialytic weight gain in response to adherence interventions. The mean difference is measured in Kg of body weight.

**Fig 5:**
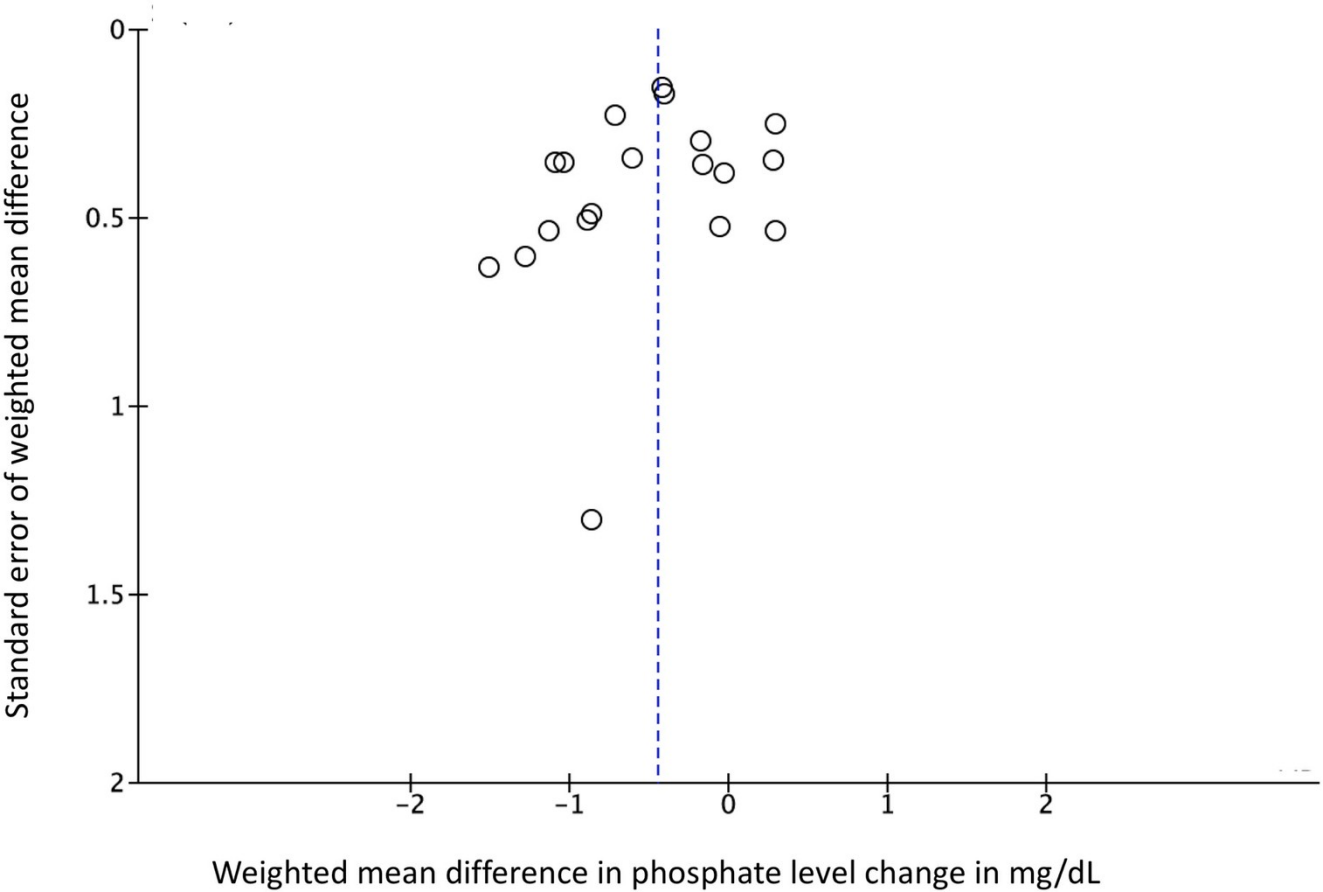
Funnel plot evaluating publication bias for studies that reported a change in phosphate level as an adherence outcome.

**Fig 6:**
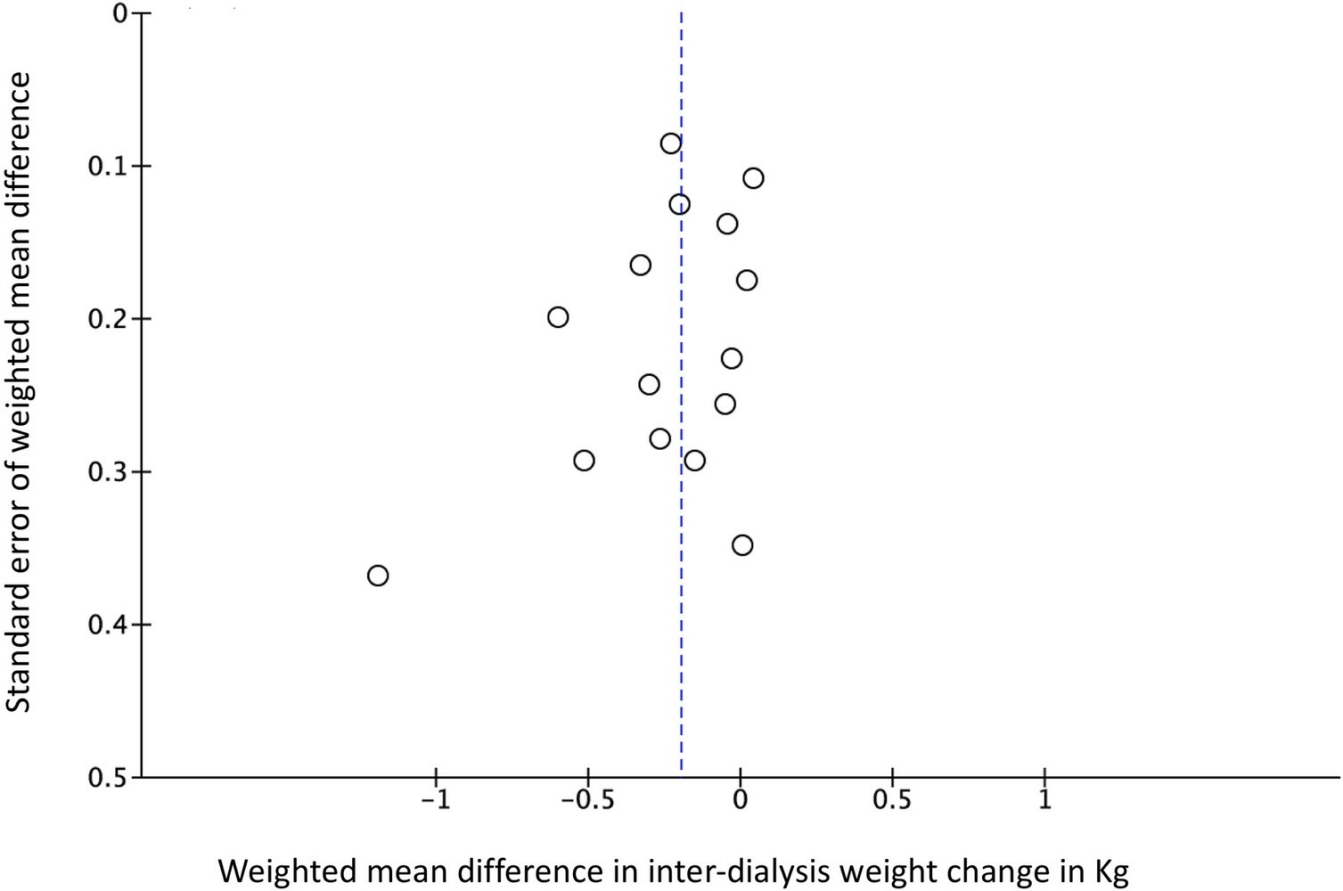
Funnel plot evaluating publication bias for studies that reported a change in inter-dialytic weight gain as an adherence outcome.

We computed pooled estimates of the mean differences in phosphate levels and inter-dialytic weight gain in response to the adherence intervention between active and control groups. Though these are only surrogate outcome measures and susceptible to confounding, we chose them for meta-analysis because these were the most commonly reported efficacy measures in the included trials. As shown in Figs 3 and 4, both phosphate levels and inter-dialytic weight gain significantly improved in response to the adherence intervention, and the level of statistical heterogeneity was moderate (I^2^ of 47% and 37% respectively) for the two analyses. As shown in Figs 5 and 6, the funnel plots did not show significant publication bias in the analysis of nineteen studies reporting a change in phosphate levels (Egger’s test p = 0.901, intercept 0.1, slope -0.33) or fifteen studies reporting a change in inter-dialytic weight gain (Egger’s test p = 0.224, intercept -1.3, slope 0.04) as the adherence outcome. Please refer to S1 and S2 Figs.

### Duration of intervention and follow-up

The median duration of total follow-up of all the included studies was 129 days, with a minimum of 4 weeks to a maximum of 12 months. The intervention could be as brief as a single educational session lasting less than an hour [13, 24] but more typically involved several structured sessions over many weeks. Educational, behavioural and psychological interventions were nearly always conducted on dialysis days in patients on haemodialysis. The duration of follow-up after completion of the intervention varied from 0 days (where the intervention continued until the final outcome assessment) to one year with a median of 42 days. For details of the duration of intervention and total follow-up of individual studies, please refer to Table 1.

Information on persistence of efficacy of intervention beyond the first outcome assessment was not provided in 20 (56%) studies. Among the remaining 16 studies, the benefits of the trialled intervention had waned or was not detectable by six weeks in one study [32] three months in four studies [8, 10, 27, 43], six months in two studies [35, 38] and by nine months [30] and twelve months [25] in one study each. The impact of the adherence intervention on the outcomes persisted for twelve months after the intervention in two trials [48, 49], for six months in two trials [34, 46], for three months in two trials [41, 50] and for one month in one trial [9]. For details of the persistence or decline of efficacy of intervention during follow-up of the individual trials, please refer to Table 1.

### Quality of evidence

Confidence in the evidence presented in this review, rated using the GRADE approach was ‘very low’ for the two most commonly reported outcomes. With respect to the factors affecting quality of evidence, we rated down the quality by one level for the domains of risk of bias, inconsistency and imprecision, while we rated down the quality by two levels for indirectness. Publication bias was not detected and did not contribute to the very low certainty of evidence. The summary of findings for these comparisons is given below in Table 2.

**Table 2:** Interventions to improve treatment adherence compared to usual care or alternative interventions for improving surrogate adherence outcomes in dialysis patients.

**Patient or population**: haemodialysis or peritoneal dialysis **Setting**: dialysis units or outpatients **Intervention**: Interventions to improve treatment adherence **Comparison**: usual care or alternative interventions					
**Outcomes**	**No of participants** **(studies)**	**Certainty of the evidence** **(GRADE)**		**Anticipated absolute effects**	
				**Risk with usual care or alternative interventions**	**Risk difference with Interventions to improve treatment adherence**
Change in inter-dialytic weight gain	1539 (15 RCTs)		⨁◯◯◯ VERY LOW ^a^^,^^b^^,^^c^^,^^d^	The mean change in inter-dialysis weight gain was **0.15 to -0.9 Kg**	MD **0.2 lower** (0.32 lower to 0.08 lower)
Change in serum phosphorous	1631 (19 RCTs)		⨁◯◯◯ VERY LOW ^a^^,^^b^^,^^c^^,^^d^	The mean change in serum phosphate was **0.59 to -1.09 mg/dL**	MD **0.45 lower** (0.66 lower to 0.23 lower)
*The risk in the intervention group (and its 95% confidence interval) is based on the assumed risk in the comparison group and the **relative effect** of the intervention (and its 95% CI). **CI:** Confidence interval; **MD:** Mean difference					
**GRADE Working Group grades of evidence** ⨁⨁⨁⨁ **High certainty:** We are very confident that the true effect lies close to that of the estimate of the effect ⨁⨁⨁◯ **Moderate certainty:** We are moderately confident in the effect estimate: The true effect is likely to be close to the estimate of the effect, but there is a possibility that it is substantially different ⨁⨁◯◯ **Low certainty:** Our confidence in the effect estimate is limited: The true effect may be substantially different from the estimate of the effect ⨁◯◯◯ **Very low certainty:** We have very little confidence in the effect estimate: The true effect is likely to be substantially different from the estimate of effect					

Explanations

a. Majority comparisons were unblinded, not analysed as intention to treat and inadequately reported important confounders and outcomes

b. Moderate heterogeneity across studies, which is not directly explained by variations in study characteristics

c. Interventions were highly diverse, used surrogate outcome measures and most trials did not undertake direct measurement of adherence

d. Optimal information size criteria unlikely to be met since the clinical importance of the pooled estimate of mean difference is doubtful

## Discussion

Our systematic review of randomized intervention trials to improve treatment adherence in dialysis patients shows a moderate, but often partial and short-lasting improvement in pre-specified adherence outcomes. The review also demonstrates a narrow focus on strategies addressing patient related factors, with shortfalls in study design and implementation, some of which are inherent to the non-discrete and heterogeneous nature of adherence behaviour.

Comprehensive adherence interventions should target the patient, the provider and the health care system and address social-economic factors, therapy related, patient related, disease related and health system related factors [1]. More than 90% of the trials included in our review addressed patient related factors contributing to non-adherence. In dialysis patients, several factors such as access to care, complexity of the treatment regimens, heavy pill burden, lower socio-economic status, poor health literacy and associated comorbidities, such as depression and cognitive impairment, can predispose to non-adherence [52]. However health care providers often erroneously assume that the patients should be motivated to adhere to the best practice treatment protocol [1], which might explain why the vast majority of trials have addressed patient related factors. It is important to recognize that the health behaviour of treatment adherence, is the product of diverse but overlapping variables, and comprehensive strategies addressing different issues are required to achieve sustained improvement in adherence.

Change in behaviour such as adherence is a very complex process, parts of which have been conceptualized in various theoretical models of behaviour in over 40% of the included trials. Not surprisingly, trials implemented by psychologists were more likely to invoke such theoretical frameworks including ‘social cognitive theory’, ‘stages of change or trans-theoretical model’, health belief model’ and ‘goal setting theory’. The integrated model of behaviour change or I-change model, which loosely assimilates the above theories and the ‘theory of planned behaviour’, assumes three phases in the process of change, namely awareness, motivation and action [53]. Information or knowledge about the various aspects of therapy is essential to build awareness, but information by itself is not sufficient to achieve or sustain behaviour change [1]. Educational or cognitive interventions constituted the sole trialled regimen in one third of the studies in this review, while they were part of the components of the interventions in the majority of studies. Improved knowledge as a result of educational interventions did not translate to a sustained improvement in the measure of adherence, at least in some studies [37, 45], confirming that behaviour change requires more than the acquisition of new knowledge.

Many of the RCTs in our review have evaluated varying combinations of educational-cognitive, behavioural-counselling and psychological-affective interventions, with significant overlap between categories. Cognitive behaviour therapy (CBT) refers to a set of intervention strategies aimed at assisting patients in identifying and altering their own dysfunctional cognitions like unhelpful thoughts, beliefs and attitudes, thereby improving their mental well-being and coping behaviour [41]. In contrast to some short-sighted behavioural modification techniques, a change in belief might enable patients to internalize the cognitive rationale for altering their behaviour [27]. CBT is a focused psychotherapy aimed at changing the way the individual thinks, feels and behaves, and, unlike traditional psychotherapy it is practical and action-oriented [10]. The systematic review on interventions to improve adherence in dialysis patients by Matteson et al, [4] concluded that cognitive behavioural interventions offered the best promise for future trials. In our review, the category of intervention, whether cognitive or behavioural or affective, was not significantly associated with the efficacy of outcome, but the overlap between categories may have affected the reliability of our analysis.

A major issue noted in this review was the frequent use of surrogate outcome measures of adherence. Biochemical and physiological measurements such as phosphate, albumin, Kt/V and inter-dialytic weight gain have been widely used as measures of adherence in dialysis patients [4, 54]. Some of these measures can be modified by factors other than adherence, like residual renal function and quality of dialysis [5]. For example, phosphate levels, which may reflect dietary non-adherence or non-adherence to phosphate binding medications may be affected by inadequate dialysis due to suboptimal vascular access. Marked variations in dialytic phosphate clearance, inconsistency in dietary phosphate absorption, and up to two-fold variations in the efficacy of phosphate binders between individuals, may account for the hyperphosphataemia in dialysis patients, rather than non-adherence to diet and medication [55]. Similarly, inter-dialytic weight gain may be unreliable as a measure of fluid non-adherence in patients with significant residual urine volume. Failure to address these confounders can lead to misclassification of adherence, and biased efficacy estimates. In this review, self-reported measures of adherence, which are well known to overestimate adherence [5] were used in one in five studies. However, direct measurement of adherence using ‘pill count’ or the electronic medication event monitoring system (MEMS) which are more robust methods to assess medication adherence [1], was used in only one out of 36 included trials.

The reporting of a large number of heterogeneous outcomes, many of which are not necessarily patient centred, is not confined to adherence intervention trials but is common in the broader nephrology literature. Initiatives are already underway to identify and implement a set of core outcomes for all trials in haemodialysis (SONG-HD) [56] and peritoneal dialysis (SONG-PD) [57] patients, based on the shared priorities of all stakeholders. The widespread adoption of standardized outcome reporting in the future will facilitate meaningful comparisons and pooling of results of dialysis patient trials, including studies evaluating treatment adherence.

A large proportion of the interventions evaluating patient related factors in this review resulted in significant improvement in some of the pre-specified measures of adherence while not impacting on other measures. Since the adherence interventions were not specific for one type of measure over the other, the observed benefits should be interpreted with caution. It is always possible that despite the interventions being outcome non-specific, some of the outcome measures, owing to their biological characteristics, may be more amenable to manipulation than others. We should also remember that when multiple comparisons are made between a study factor and several outcome factors, some of them may return a significant effect due to chance alone.

In a chronic disease like ESKD, it would be reasonable to assume that persistent long-term adherence to therapy would be required to achieve sustained clinical benefits. In our review, the follow-up period for the adherence interventions was relatively short, ranging from four weeks to 12 months. It is conceivable that in patients with ESKD, trials with such short follow-up periods are unlikely to identify any meaningful clinical benefits, other than surrogate markers of adherence which demonstrate a proof of concept about the efficacy of adherence interventions.

Even when a behavioural change in response to an adherence intervention is adopted, relapse of the original behaviour of non-adherence can occur and relapse prevention is an important strategy in achieving long-term adherence [37]. With brief interventions and short duration of follow-up, there is less opportunity for behavioural reinforcement, with a higher attendant risk of relapse of non-adherence. The majority of the studies (55%) in our review did not report whether adherence persisted beyond the first outcome assessment. In the sixteen studies that provided this information the beneficial effect of adherence intervention waned or was not detectable by 12 months or less in nine studies, while a persistent benefit at 12 months was reported in only two studies. These observations also highlight the importance of having a longer duration of adherence interventions, with appropriate reinforcement strategies, and longer periods of follow-up, to help demonstrate the sustained benefit from such interventions.

Our review has several strengths. It summarizes the research that addresses an important aspect of dialysis patient care, which has the potential to improve patient outcomes. The broad criteria for including trials in this review helped us to cover all important domains of dialysis patient therapy. This has also helped us to identify a significant number of studies including those used in the meta-analysis. Presenting the breadth of categories of adherence interventions and outcomes, in both haemodialysis and peritoneal dialysis patients, based on up to date medical literature, is the main contribution of the current review to the existing research in this field.

However, this review has several limitations. Many of the included trials were small in size, took place in diverse clinical settings and were not of a high methodological quality. The interventions were not homogeneous in nature or intensity. There was a lack of consistency in outcome reporting and follow-up between studies. This made it difficult to effectively compare the interventions or recommend specific strategies to improve treatment adherence in this vulnerable population. The widespread use of surrogate adherence efficacy measures, with the potential for residual confounding, and the relatively short duration of follow-up, made it difficult to judge the true extent of sustainable benefit from these interventions. It is noteworthy that only 8% of the included trials recruited patients on peritoneal dialysis, which could limit the applicability of our review to this population. We included studies which were published as full text articles in English language only and may have missed important trials published in non-English languages. Including such trials may have improved the quality of our review, but this was out of the scope of resources available for this project.

The quality of evidence in this review was rated as ‘very low’ using the GRADE approach. This would imply that the true effect may be substantially different from the effect estimate detected in our review and future high-quality studies may provide different results. The insights from conducting this review enable us to propose, what such a high-quality adherence intervention trial should aim to achieve. The interventions should be well defined and translatable to routine clinical practice. The outcomes should be clinically meaningful, and the study design should specifically address factors other than adherence which can confound the evaluated outcome measures, especially when surrogate measures of adherence efficacy are used. Direct measurement of adherence should be undertaken whenever feasible. The intervention should be of sufficient duration with a plan for periodic reinforcement, which is essential for sustained behaviour change. The follow-up should extend beyond intervention to assess its residual effect and identify the risk of relapse of non-adherence. With an increased interest in the topic in recent years, it is realistic to anticipate that such robust trials will emerge and the results from such trials will enable us to identify effective adherence interventions that would become part of routine clinical care in the future.

## Conclusions

In this systematic review we have identified that interventions to improve treatment adherence in dialysis patients are effective in improving surrogate efficacy outcomes, at least in the short term. However, there is considerable scope for improvement in the design and conduct of adherence intervention trials to evaluate whether specific strategies can lead to reliable and lasting benefits with respect to meaningful clinical outcomes in patients with ESKD who are on dialysis.

## Supporting information

S1 Table Example literature search strategy for medline used in the review. (DOCX)

S2 Table Data extraction matrix providing detailed information on study characteristics. (XLSX)

S3 Table Risk of bias assessment of individual studies included in the review. (XLSX)

S1 Fig Egger’s publication bias plot for trials evaluating change in phosphate levels. (TIF)

S2 Fig Egger’s publication bias plot for trials evaluating change in inter-dialytic weight gain. (TIF)

S1 File PRISMA checklist. (DOCX)
