# Supplementary material for: Strategies to improve dietary, fluid, dialysis or medication adherence in patients with end stage kidney disease on dialysis: A systematic review and meta-analysis of randomized intervention trials
Source: PLoS One. 2019 Jan 29;14(1):e0211479. doi: 10.1371/journal.pone.0211479 (PMC6350978; doi:10.1371/journal.pone.0211479)
Supplement: S1 Table — (DOCX) [file pone.0211479.s001.docx]

**S1 Table. Example search strategy (PubMed on 01/07/2018)**

| ID | Search |
| --- | --- |
| # 1 | exp Dialysis (MeSH term) |
| # 2 | exp Renal dialysis (MeSH term) |
| # 3 | exp Peritoneal dialysis, continuous ambulatory (MeSH term) |
| # 4 | exp Peritoneal dialysis (MeSH term) |
| # 5 | dialys*/ or inter-dialy*/ or interdialy*/ or haemodialys*/ or hemodialys*/ or peritoneal dialys*/ or CAPD.mp (mapping alias or text word) |
| # 6 | # 1 or # 2 or # 3 or # 4 or # 5 |
| # 7 | exp Treatment adherence / or exp Treatment compliance / or exp Medication adherence (MeSH terms) |
| # 8 | (fluid* and adhere*) or (fluid* and non-adheren*) or (fluid* and nonadheren*) or (fluid* and complian*) or (fluid* and non-complian*) or (fluid* and noncomplian*).mp (mapping alias or text word) |
| # 9 | (diet* and adhere*) or (diet* and non-adheren*) or (diet* and nonadheren*) or (diet* and complian*) or (diet* and non-complian*) or (diet* and noncomplian*).mp (mapping alias or text word) |
| # 10 | (medication* and adhere*) or (medication* and non-adheren*) or (medication* and nonadheren*) or (medication* and complian*) or (medication* and non-complian*) or (medication* and noncomplian*).mp (mapping alias or text word) |
| # 11 | # 7 or # 8 or # 9 or # 10 |
| # 12 | # 6 and # 11 |
| # 13 | limit # 12 to (English language and humans) |
