# Supplementary figures and images for: Strategies to improve dietary, fluid, dialysis or medication adherence in patients with end stage kidney disease on dialysis: A systematic review and meta-analysis of randomized intervention trials
Source: PLoS One. 2019 Jan 29;14(1):e0211479. doi: 10.1371/journal.pone.0211479 (PMC6350978; doi:10.1371/journal.pone.0211479)

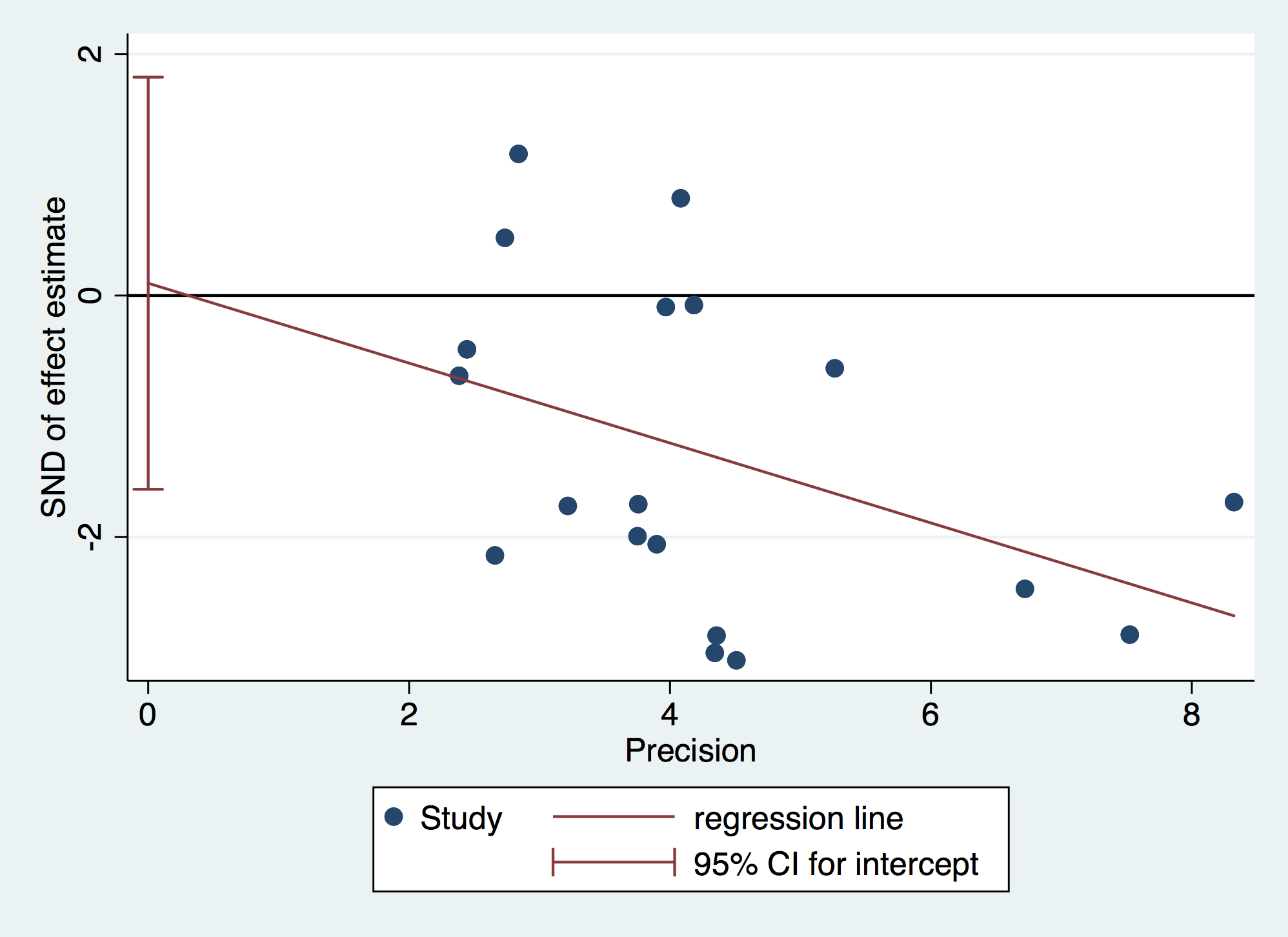

Supplement: S1 Fig — (TIF) [file pone.0211479.s004.tif]

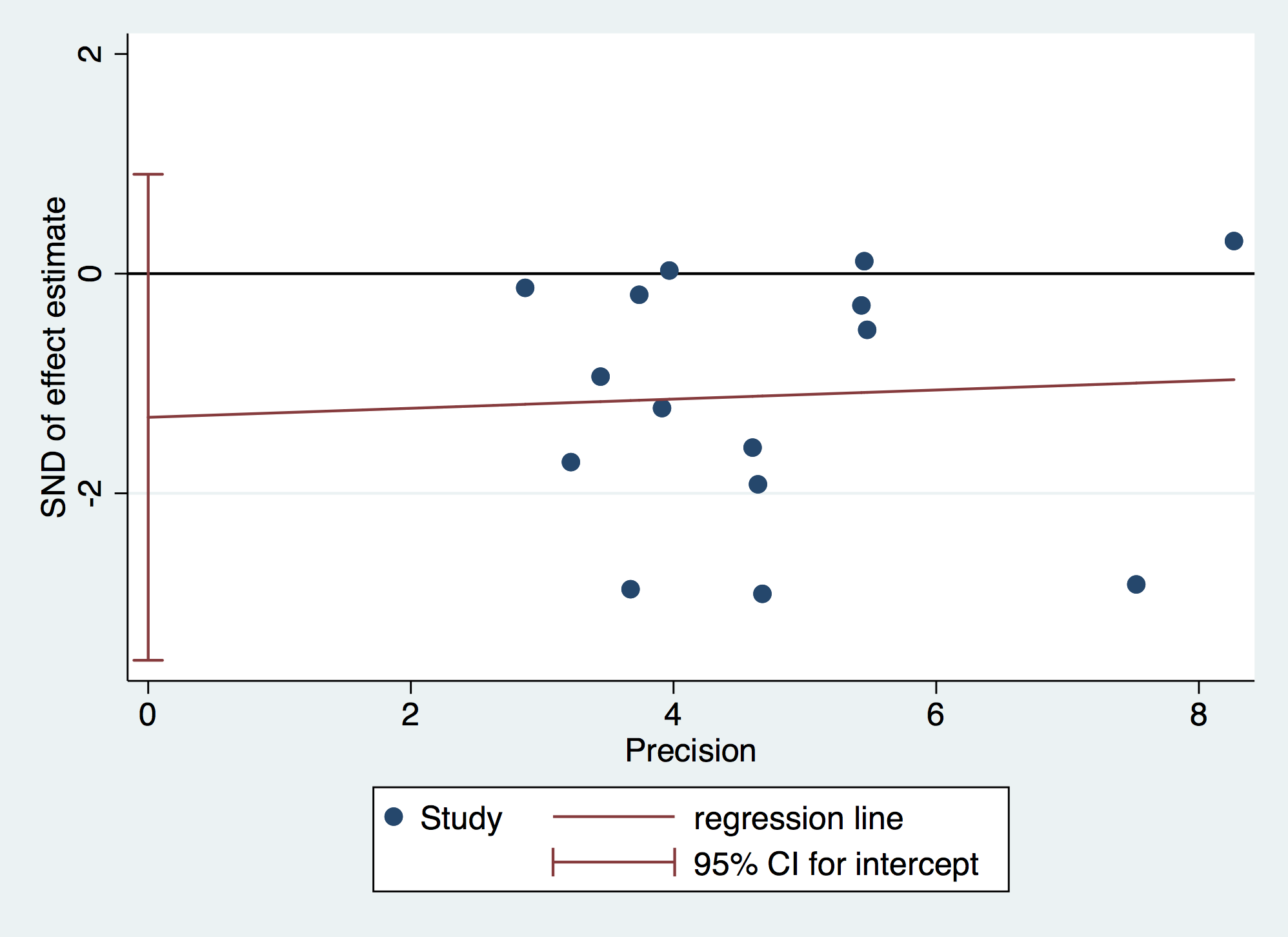

Supplement: S2 Fig — (TIF) [file pone.0211479.s005.tif]
