# Supplementary material for: Strategies to improve dietary, fluid, dialysis or medication adherence in patients with end stage kidney disease on dialysis: A systematic review and meta-analysis of randomized intervention trials
Source: PLoS One. 2019 Jan 29;14(1):e0211479. doi: 10.1371/journal.pone.0211479 (PMC6350978; doi:10.1371/journal.pone.0211479)
Supplement: S1 File — (DOCX) [file pone.0211479.s006.docx]

| **Section/topic** | **#** | **Checklist item** | **Reported on page #** |
| --- | --- | --- | --- |
| **TITLE** | | |  |
| Title | 1 | Identify the report as a systematic review, meta-analysis, or both. – Identified as a systematic review & meta-analysis | 1 |
| **ABSTRACT** | | |  |
| Structured summary | 2 | Provide a structured summary including, as applicable: background; objectives; data sources; study eligibility criteria, participants, and interventions; study appraisal and synthesis methods; results; limitations; conclusions and implications of key findings; systematic review registration number. – Abstract provides a comprehensive summary in 300 words. | 2-3 |
| **INTRODUCTION** | | |  |
| Rationale | 3 | Describe the rationale for the review in the context of what is already known. – Given in section of introduction | 3-4 |
| Objectives | 4 | Provide an explicit statement of questions being addressed with reference to participants, interventions, comparisons, outcomes, and study design (PICOS). – PICO statement and study objectives given at the end of introduction | 4-5 |
| **METHODS** | | |  |
| Protocol and registration | 5 | Indicate if a review protocol exists, if and where it can be accessed (e.g., Web address), and, if available, provide registration information including registration number. The review was prospectively registered with PROSPERO in February 2018- CRD42018087899, details given in the method section of manuscript. | 5 |
| Eligibility criteria | 6 | Specify study characteristics (e.g., PICOS, length of follow-up) and report characteristics (e.g., years considered, language, publication status) used as criteria for eligibility, giving rationale. – Provided in manuscript in detail | 5-6 |
| Information sources | 7 | Describe all information sources (e.g., databases with dates of coverage, contact with study authors to identify additional studies) in the search and date last searched. – Medline, Embase, Cochrane register of randomized trials and hand-search. Details are given in the manuscript. | 5-6 |
| Search | 8 | Present full electronic search strategy for at least one database, including any limits used, such that it could be repeated. – Medline search strategy example provided as supporting information S1 Table. Details of search terms used given in manuscript pages 5-6. Literature search finalized on 1^st^ July 2018. No limit on publication years were set. Search limited to English language and humans. | 5-6, S1 Table |
| Study selection | 9 | State the process for selecting studies (i.e., screening, eligibility, included in systematic review, and, if applicable, included in the meta-analysis). – Provided in detail in the manuscript | 5-6, 9 |
| Data collection process | 10 | Describe method of data extraction from reports (e.g., piloted forms, independently, in duplicate) and any processes for obtaining and confirming data from investigators. – standard check list developed by authors used. Items in the check list has been outlined in the manuscript. | 6-7 |
| Data items | 11 | List and define all variables for which data were sought (e.g., PICOS, funding sources) and any assumptions and simplifications made. – Authors were contacted about missing information in the paper to facilitate meta-analysis. This is described in the section on statistics. Assumptions used in meta-analysis - estimation of standard deviation from p values or imputing from arithematic mean, if not available in the paper. Details given in manuscript | 6-8 |
| Risk of bias in individual studies | 12 | Describe methods used for assessing risk of bias of individual studies (including specification of whether this was done at the study or outcome level), and how this information is to be used in any data synthesis. – Risk of bias estimated using the Cochrane Risk of bias tool 2.0. Risk of bias was assessed for the main outcome for each study by two authors independently and inter-rater agreement was assessed using Cohen’s kappa. | 7-8, 13 |
| Summary measures | 13 | State the principal summary measures (e.g., risk ratio, difference in means). Mean difference between intervention and control arms for the summary measures used in meta-analysis | 8 |
| Synthesis of results | 14 | Describe the methods of handling data and combining results of studies, if done, including measures of consistency (e.g., I^2^) for each meta-analysis.). Meta-analysis done using Revman, Forest plots created, Heterogeneity assessed by I^2^ statistics using Revman and publication bias assessed by Funnel plots and Egger’s test. | 8 |

Page 1 of 2

| **Section/topic** | **#** | **Checklist item** | **Reported on page #** |
| --- | --- | --- | --- |
| Risk of bias across studies | 15 | Specify any assessment of risk of bias that may affect the cumulative evidence (e.g., publication bias, selective reporting within studies). – Selective reporting assessed as per ROB tool, publication bias assessed by Funnel plots | 7-8 |
| Additional analyses | 16 | Describe methods of additional analyses (e.g., sensitivity or subgroup analyses, meta-regression), if done, indicating which were pre-specified. – Not applicable |  |
| **RESULTS** | | |  |
| Study selection | 17 | Give numbers of studies screened, assessed for eligibility, and included in the review, with reasons for exclusions at each stage, ideally with a flow diagram. | 9 & Fig 1 |
| Study characteristics | 18 | For each study, present characteristics for which data were extracted (e.g., study size, PICOS, follow-up period) and provide the citations. Data abstraction done as described in the methods. Key study characteristics are given in Table 1 and more details are provided in S1 Table. | 10-12, Table 1, S1 Table |
| Risk of bias within studies | 19 | Present data on risk of bias of each study and, if available, any outcome level assessment (see item 12). Risk of bias assessment of the individual domains provided as Fig 2. Details of risk of bias assessment of individual studies with rationale for such assessment is given in S3 Table. | 13-14, Fig 2, S3 Table |
| Results of individual studies | 20 | For all outcomes considered (benefits or harms), present, for each study: (a) simple summary data for each intervention group (b) effect estimates and confidence intervals, ideally with a forest plot. – Important results are provided in the result section of manuscript and key details given in Table 1. Meta-analysis results given as Forest plots for the two most common outcomes. | 14-18, Fig 3, Fig 4 |
| Synthesis of results | 21 | Present results of each meta-analysis done, including confidence intervals and measures of consistency. – Forest plots provide information of confidence intervals and I^2^ to assess heterogeneity. GRADE assessment was done to assess the quality of the review. | 17-18, Fig 3, Fig 4, |
| Risk of bias across studies | 22 | Present results of any assessment of risk of bias across studies (see Item 15). – Funnel plots to assess publication bias and Egger’s test to objectively assess publication bias using regression. | 17, Fig 2, 5 & 6, S1 Fig, S2 Fig |
| Additional analysis | 23 | Give results of additional analyses, if done (e.g., sensitivity or subgroup analyses, meta-regression [see Item 16]). – Not applicable |  |
| **DISCUSSION** | | |  |
| Summary of evidence | 24 | Summarize the main findings including the strength of evidence for each main outcome; consider their relevance to key groups (e.g., healthcare providers, users, and policy makers). Detailed discussion about the findings and applicability and recommendations for future research provided. | 20-25 |
| Limitations | 25 | Discuss limitations at study and outcome level (e.g., risk of bias), and at review-level (e.g., incomplete retrieval of identified research, reporting bias). Detailed discussion on limitations provided | 24 |
| Conclusions | 26 | Provide a general interpretation of the results in the context of other evidence, and implications for future research. This is provided in the concluding section of discussions and conclusions. | 24-26 |
| **FUNDING** | | |  |
| Funding | 27 | Describe sources of funding for the systematic review and other support (e.g., supply of data); role of funders for the systematic review. – Declaration given at the end of the manuscript after references | 30 |

*From:*  Moher D, Liberati A, Tetzlaff J, Altman DG, The PRISMA Group (2009). Preferred Reporting Items for Systematic Reviews and Meta-Analyses: The PRISMA Statement. PLoS Med 6(7): e1000097. doi:10.1371/journal.pmed1000097

For more information, visit: **www.prisma-statement.org**.

Page 2 of 2
